# Editorial: Diagnosis and Treatment of Breast Cancer in 2022: The Rise of Novel Molecular Biomarkers

**DOI:** 10.3389/fmolb.2022.1117323

**Published:** 2023-01-04

**Authors:** Nicola Fusco, Umberto Malapelle, Carmen Criscitiello

**Affiliations:** ^1^ Department of Oncology and Hemato-Oncology, University of Milan, Milan, Italy; ^2^ Division of Pathology, IEO, European Institute of Oncology IRCCS, Milan, Italy; ^3^ Department of Public Health, Federico II University of Naples, Naples, Italy; ^4^ Division of Early Drug Development for Innovative Therapy, IEO, European Institute of Oncology, Milan, Italy

**Keywords:** breast cancer, editorial, biomarkers, predictive pathology, precision medicine

We are experiencing an amazing era of advances in the diagnosis and treatment of breast cancer, with the discovery of novel actionable biomarkers (e.g., programmed death-ligand 1 (PD-L1), phosphatidylinositol-4,5-bisphosphate 3-kinase, catalytic subunit alpha (*PIK3CA*), mismatch repair) and the re-discovery of traditional and established ones, particularly for HER2 (low expression/mutations) (Tarantino et al., 2020; Fusco et al., 2021a; Sajjadi et al., 2021; Criscitiello et al., 2022). These biomarkers are strongly impacting pathology and treatment decision-making in oncology, with the introduction of ultra-personalized therapeutic options (Punturi et al., 2021; Venetis et al., 2022a; Henry et al., 2022; Tarantino et al., 2022). In this evolving scenario, predictive molecular pathology is called to face new challenges in breast cancer (Fusco et al., 2021b; Dileep and Gianchandani Gyani, 2022). Our improved diagnostic resolution, together with the combination of clinicopathologic data and massive molecular and digital data, is allowing targeted therapies to become more and more selective (Pisapia et al., 2022). The multi-dimension of this approach requires extremely precise testing methods and guidelines.

We edited the present Research Topic of Frontiers in Molecular Biosciences on The Rise of Novel Molecular Biomarkers in Breast cancer (2022 edition) to provide a snapshot of novel significant advances in the evolving field of breast cancer biomarkers. We selected two original research articles and two comprehensive reviews covering different aspects of biomarkers in breast cancer. Among these, an original research article by Elham Sajjadi and collaborators provides previously unavailable evidence on the biology underpinning invasive breast carcinomas with osteoclast-like stromal giant cells (OSGC) (Sajjadi et al., 2022). Through a comprehensive characterization of the different cellular compartments of this exceedingly rare type of tumor, the Authors investigated the similarities of OSGC with tumor and tumor immune microenvironment in terms of morphology, protein, and monocytic miRNA signatures. This comprehensive approach unveiled shared epigenetic events during the ontogenesis of breast cancer cells and OSGC, with the latter belonging to the spectrum of M2 tumor-associated macrophages. Another mRNA study by Chen et al. Depicts the prognostic value and biological role of protein tyrosine kinase 2 (PTK2) in breast cancer Chen et al. This elegant work has been particularly welcomed by the Editorial Board, given the integration of multiple publicly available databases. The Authors found that PTK2 mRNA is upregulated in breast cancer cells compared to the normal breast tissue and that it may give significant prognostic information, being associated with high-stage, mutations in well-known cancer genes, and poor survival. Quality of life Research Topic is a crucial burden in breast cancer survivors and there is accumulating evidence on the role of biomarkers in this spectrum of conditions (Maurer et al., 2021; Sunilkumar et al., 2021; Invernizzi et al.). In this respect, a multicentric Italian effort led by Prof. Marco Invernizzi clarifies the current state of knowledge on biomarkers that might be potentially integrated into rehabilitation practice to promote a precision medicine approach to breast cancer-specific survivorship Research Topic Invernizzi et al. This systematic review, which includes 22 randomized controlled trials assessing rehabilitation interventions in patients with breast cancer, reveals that physical exercise in patients with a diagnosis of breast cancer induce the presence of recurrent molecular alterations in key molecules, thus corroborating the need for translational research focused on the role of biomarkers in tailored rehabilitative treatments in these women. Finally, we are delighted to offer to the readership of this Research Topic some real-world data by Di Cosimo S. et al. On the prevalence, treatment response, and outcome of patients with HER2-low breast cancer Di Cosimo et al. This study is of particular relevance given that the introduction of novel anti-HER2 compounds is determining a paradigm shift in breast cancer treatment, as tumors with low levels of HER2 expression (i.e., score 1+/2+ with no gene amplification) benefit from HER2 antibody-drug conjugates (ADC) (Venetis et al., 2022b). In this article, patients with HER2-low breast cancer account for ∼50% of the cases treated with neoadjuvant therapy and have poor treatment responses. In the absence of pathologic complete response (pCR), these patients, particularly those with a triple-negative neoplasm, have a dismal prognosis. Studies are needed not only to better define the biology of HER2-low breast cancer but also to investigate the possible role of new therapeutic approaches in the early stage.

Our understanding of the molecular landscape of breast cancer is expanding day by day. We believe that a biomarker-based approach being able to identify specific subgroups of patients is now mandatory in breast cancer precision oncology. Hence, precision medicine should ideally start in the pathology lab and expand to the clinic, keeping the patients at the center of their management.

## References

[B1] Criscitiello C. Guerini-Rocco E. Viale G. Fumagalli C. Sajjadi E. Venetis K. (2022). Immunotherapy in breast cancer patients: A focus on the use of the currently available biomarkers in oncology. Anticancer Agents Med. Chem. 22 (4), 787–800. 10.2174/1871520621666210706144112 34229592

[B2] Dileep G. Gianchandani Gyani S. G. (2022). Artificial intelligence in breast cancer screening and diagnosis. Cureus 14 (10), e30318. 10.7759/cureus.30318 36381716 PMC9650950

[B3] Fusco N. Malapelle U. Fassan M. Marchiò C. Buglioni S. Zupo S. (2021). PIK3CA mutations as a molecular target for hormone receptor-positive, HER2-negative metastatic breast cancer. Front. Oncol. 11, 644737. 10.3389/fonc.2021.644737 33842357 PMC8027489

[B4] Fusco N. Ragazzi M. Sajjadi E. Venetis K. Piciotti R. Morganti S. (2021). Assessment of estrogen receptor low positive status in breast cancer: Implications for pathologists and oncologists. Histol. Histopathol. 36, 1235–1245. 10.14670/hh-18-376 34585734

[B5] Henry N. L. Somerfield M. R. Dayao Z. Elias A. Kalinsky K. McShane L. M. (2022). Biomarkers for systemic therapy in metastatic breast cancer: ASCO guideline update. J. Clin. Oncol. 022 (0), 3205–3221. 10.1200/jco.22.01063 35759724

[B6] Invernizzi M. de Sire A. Venetis K. Cigna E. Carda S. Borg M. (2022). Quality of life interventions in breast cancer survivors: State of the art in targeted rehabilitation strategies. Anticancer Agents Med. Chem. 22 (4), 801–810. 10.2174/1871520621666210609095602 34151769

[B7] Maurer T. Jaskulski S. Behrens S. Jung A. Y. Obi N. Johnson T. (2021). Tired of feeling tired - the role of circulating inflammatory biomarkers and long-term cancer related fatigue in breast cancer survivors. Breastedinbg. Scotl. 56, 103–109. 10.1016/j.breast.2021.02.008 PMC7937559 33668004

[B8] Pisapia P. L'Imperio V. Galuppini F. Sajjadi E. Russo A. Cerbelli B. (2022). The evolving landscape of anatomic pathology. Crit. Rev. Oncol. Hematol. 178, 103776. 10.1016/j.critrevonc.2022.103776 35934262

[B9] Punturi N. B. Seker S. Devarakonda V. Mazumder A. Kalra R. Chen C. H. (2021). Mismatch repair deficiency predicts response to HER2 blockade in HER2-negative breast cancer. Nat. Commun. 12 (1), 2940. 10.1038/s41467-021-23271-0 34011995 PMC8134423

[B10] Sajjadi E. Venetis K. Piciotti R. Invernizzi M. Guerini-Rocco E. Haricharan S. (2021). Mismatch repair-deficient hormone receptor-positive breast cancers: Biology and pathological characterization. Cancer Cell Int. 21 (1), 266. 10.1186/s12935-021-01976-y 34001143 PMC8130151

[B11] Sunilkumar M. M. Finni C. G. Lijimol A. S. Rajagopal M. R. (2021). Health-Related suffering and palliative care in breast cancer. Curr. Breast Cancer Rep. 13, 241–246. 10.1007/s12609-021-00431-1 34804375 PMC8593626

[B12] Tarantino P. Hamilton E. Tolaney S. M. Cortes J. Morganti S. Ferraro E. (2020). HER2-Low breast cancer: Pathological and clinical landscape. J. Clin. Oncol. 38 (17), 1951–1962. 10.1200/jco.19.02488 32330069

[B13] Tarantino P. Jin Q. Tayob N. Jeselsohn R. M. Schnitt S. J. Vincuilla J. (2022). Prognostic and biologic significance of ERBB2-low expression in early-stage breast cancer. JAMA Oncol. 8, 1177–1183. 10.1001/jamaoncol.2022.2286 35737367 PMC9227690

[B14] Venetis K. Pepe F. Munzone E. Sajjadi E. Russo G. Pisapia P. (2022). Analytical performance of next-generation sequencing and RT-PCR on formalin-fixed paraffin-embedded tumor tissues for PIK3CA testing in HR+/HER2− breast cancer. Cells 11 (22), 3545. 10.3390/cells11223545 36428975 PMC9688837

[B15] Venetis K. Crimini E. Sajjadi E. Corti C. Guerini-Rocco E. Viale G. (2022). HER2 low, ultra-low, and novel complementary biomarkers: Expanding the spectrum of HER2 positivity in breast cancer. Front. Mol. Biosci. 9, 834651. 10.3389/fmolb.2022.834651 35372498 PMC8965450

